# Fact or Fiction? The Development and Evaluation of a Tobacco Virtual Health Tool

**DOI:** 10.3390/ijerph20021397

**Published:** 2023-01-12

**Authors:** Geneviève Jessiman-Perreault, Rachel Dunn, Angela Erza, Candace Kratchmer, Ameera Memon, Howie Thomson, Lisa Allen Scott

**Affiliations:** 1 Provincial Population and Public Health, Alberta Health Services, Holy Cross Centre, 2210 2 St SW, Calgary, AB T2S 3C3, Canada; 2 Department of Oncology, Cumming School of Medicine, University of Calgary, 3330 Hospital Dr NW, Calgary, AB T2N 4N1, Canada; 3 Department of Community Health Sciences, Cumming School of Medicine, University of Calgary, 3330 Hospital Dr NW, Calgary, AB T2N 4N1, Canada

**Keywords:** virtual health, e-health, tobacco cessation, evaluation

## Abstract

The virtual setting is an important setting for health promotion as individuals increasingly go online for health information and support. Yet, users can have difficulty finding valid, trustworthy, and user-friendly health information online. In 2022, we launched an interactive Fact or Fiction Tobacco virtual health tool. The virtual health tool uses evidence-informed tailored content to engage users and refer them to local tobacco cessation resources. The present paper describes the development, user testing, and evaluation of this tool. The Fact or Fiction virtual health tool was designed by tobacco cessation and health marketing experts and informed by health behaviour theories of change. The tool captures data on who is seeking health information, the user’s stage of readiness to quit tobacco products, and whether they act by accessing referred resources. In 2021, we conducted two phases of user testing prior to marketing the tool publicly. After 7 weeks of marketing, we collected data on user interactions with the tool and evaluated the reach of the tool. Results from user testing found the tool to be engaging, easy to use, and quick to complete. Adaptations were made to simplify and condense text and include additional animations. During the first seven weeks of the tool being live, it reached 2306 users, and 38.7% of those users were current or occasional tobacco users. Users were classified based on their intention to quit. Bivariate analysis found that the tool was successful in driving tobacco users towards action as 21.2% tobacco users who were looking to quit and 8.8% of tobacco users who were not looking to quit clicked on local tobacco cessation resources. This virtual health tool is reaching the targeted population and providing tailored information needed at each stage of the continuum of health behaviour change. Among tobacco users looking to quit, this virtual health tool acts as a quick referral to local tobacco cessation resources.

## 1. Introduction

The past two decades have seen a surge in the use of digital technology, prompting the inclusion of virtual spaces as a new setting within the socioecological model for health promotion [1]. At the same time, we find ourselves at the forefront of a transformational shift in attitudes, as the internet has eclipsed physicians as many peoples’ first and most frequent source of health information [2]. Given the volume of information available about health issues in the online setting, it is often difficult for users, particularly those with lower health literacy, to determine which information is valid, helpful, and trustworthy [3,4]. Moreover, the spread of misinformation about health issues in online spaces is a public health crisis that has profound impacts on attitudes and behaviours towards risk factors for chronic diseases [5].

There is an urgent need to identify ‘where individuals are at’ regarding their knowledge about modifiable risk factors for cancer and chronic disease and then effectively respond by delivering trustworthy, evidence-informed, actionable information in the digital health setting. Through customized content to meet individuals ‘where they are at’, virtual health tools can provide users who are ready with the curated resources they need to take action to improve their health by addressing modifiable risk factors.

Virtual health tools have often been applied to health behaviour change by combining theories of health psychology to motivate and encourage behaviour change and health informatics to engage users in the platform and tailor content to their needs [6,7]. A growing body of research has been conducted on the potential of virtual screening, brief intervention, and referral for treatment (SBIRT) for addressing alcohol and drug use [8] and maternal health [9]. Yet, these tools are often implemented in the healthcare setting and are not directly accessible to individuals looking to improve their health. Moreover, despite promising evidence for the effectiveness of SBIRT for tobacco use delivered in-person in primary care settings [10], there are few examples of the implementation of such a strategy in the virtual setting.

The effectiveness of applying persuasive design techniques to virtual health tools is supported by a recent review of 85 studies that reported positive health and wellness changes in 75% of studies included [11]. The use of virtual health tools can have many positive health outcomes, but those outcomes often rely on the ease of use for end-users. To determine ease of use, these tools must undergo extensive testing to assess usability [12]. Despite the rapid growth in the development and implementation of virtual health tools, there is a paucity of studies that describe and report outcomes from process and usability evaluations [13].

Here, we present a detailed description of the content development, including usability testing, of a virtual health tool focused on tobacco use—the Fact or Fiction Tobacco tool. The objective of the Tobacco Fact or Fiction tool is to motivate users to consider quitting using tobacco products and increase users’ knowledge about the supports available to them to overcome the barriers that might be keeping them from taking action to quit smoking. Informed by the RE-AIM evaluation framework [14], we will present results from the evaluation of the first 7 weeks of data collected from the tool in alignment with the Reach indicator [14]. Specifically, we will present results from the number of users of the Fact or Fiction Tobacco tool and what percentage of users are current or occasional tobacco users. Finally, we assess whether the percentage of users who click on the recommended supports for tobacco cessation or setting-based health promotion differed by their tobacco use status and stage of change.

## 2. Materials and Methods

### 2.1. Virtual Tool Conceptual Development

**Figure 1:**
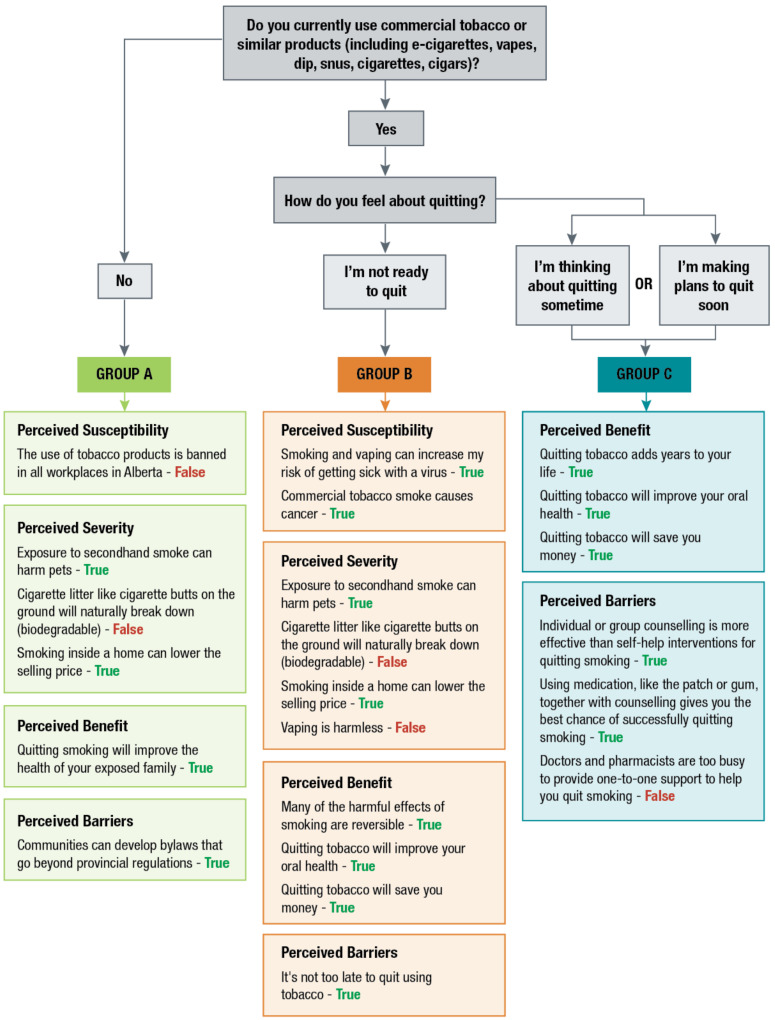
Fact or Fiction Tobacco Conceptual Model.

The Fact or Fiction Tobacco tool aimed to fill the information needs of users looking to quit using tobacco by motivating individuals to continue to attempt to quit using tobacco products, informing them of available local resources that can assist them in their cessation journey, and finally connecting them with those local resources for support. To this end, a cancer prevention research team embedded within Alberta Health Services (AHS) partnered with tobacco cessation experts who deliver a provincial tobacco cessation program (AlbertaQuits and QuitCore) to create a virtual health promotion and referral tool for tobacco cessation. The Fact or Fiction Tobacco tool presents users with a series of five statements that are either based on evidence or falsehoods. Falsehoods represent common myths and misconceptions on that health topic. As each statement is presented, the user will be asked to identify whether the statement constitutes ‘fact’ or ‘fiction’. These statements were designed by subject area experts within AHS and members of our research team. Fifteen statements were developed based on the Health Belief Model (HBM) to align with each construct of the theory (perceived susceptibility, perceived severity, perceived benefits, and perceived barriers) (see Figure 1 for final statements for Group A, B, and C) [15].

In the Fact or Fiction Tobacco tool, the user is presented with fact or fiction statements dependent on responses to two or three screening questions that determine whether they are current or occasional tobacco users and whether (and when) they intend to quit using tobacco products. To determine users’ tobacco use status, individuals are first asked, “Do you currently use commercial tobacco or similar products (including e-cigarettes, vapes, dip, snus, cigarettes, cigars)?”. If the user selects “yes”, they are asked, “How do you feel about quitting” and are given three response options (“I am not ready to quit”, “I am thinking about quitting”, “I am making plans to quit”). If the user selects “no”, they are asked, “Is someone in your family looking to quit using tobacco?”. Based on responses to these screening questions, users of the tool were classified as non-tobacco users (Group A), tobacco users in the pre-contemplation stage (i.e., not looking to quit) (Group B), and tobacco users in the contemplation stage (i.e., looking to quit) (Group C), based on the Transtheoretical Model (TTM) [16].

The TTM posits that health behaviour change is a process that unfolds over time and that individuals at each stage require different information, resources, and support to progress through the stages towards health behaviour change action [16]. By pairing the HBM and the TTM theoretical models, we designed the tool to present different statements depending on the stage of change of the user (see Figure 1). Users of the tool who are current tobacco users but do not intend to quit soon are classified as pre-contemplation (Group B) and are provided with statements that emphasize the perceived severity, perceived benefits, and perceived self-efficacy of tobacco cessation to increase their motivation to quit in the next 6 months. Users of the tool who are current tobacco users but do intend to quit soon are classified as contemplation (Group C) and are presented with statements that focus on perceived self-efficacy and perceived barriers to increase their knowledge about the supports available to them to overcome the barriers that might be keeping them from taking action. Individuals in the contemplation stage are aware of the harms associated with tobacco use and the benefits of quitting and are therefore not presented with statements on the perceived severity or perceived benefits of tobacco use. Users who are not current tobacco users (Group A) are provided with statements focused on how they can become advocates for smoke-free workplaces and communities and how they can support their family and friends to quit using tobacco products.

After providing an answer to each statement, the user interacting with the tool will be told whether they answered the question correctly or incorrectly, and the individual will be provided with additional information that elaborates on the “fact” or explains why the statement is a falsehood. In addition, users who identify themselves as current or occasional tobacco users in the screening questions (Group B and Group C) will be presented with links to evidence-based programs and resources provided by our AHS partners (i.e., AlbertaQuits and QuitCore). These links act as indicators of user intention to take action to improve their health by accessing support to help them quit using tobacco. At the end of the tool, users were provided with (1) their score; (2) a message designed to encourage them to modify their health behaviour; and (3) a link to connect the user with tobacco cessation-focused resources (i.e., links to AlbertaQuits and QuitCore or to enter their postal code to be connected with resources in their area) or settings- (e.g., workplace and community) based health promotion resources.

### 2.2. User Testing

Two phases of user testing were undertaken to inform the final design and delivery of the Tobacco Fact or Fiction tool. The first phase (Phase 1) of user testing focused on examining the content and “look and feel” of the Fact or Fiction Tobacco statements and imagery.

For the first phase of user testing, a survey was developed, programmed into RedCap software, and distributed in August 2021 to a panel of individuals (*n* = 258) who consented to provide feedback on AHS programs and communications. The survey contained screenshots from the design of the tool and featured completed images and text. Respondents were asked to read the statement, examine the images, and respond to a few short questions (see Appendix A for the questionnaire). Given the multiple branches of the Fact or Fiction tool, user testing participants were asked whether they are current or occasional tobacco users and if so, whether they are planning to quit. This ensured respondents received the correct branch as they would have done if they were using the live Fact or Fiction tool and allowed us to tailor the survey questions to the respondents’ tobacco use status.

After implementing the suggested changes from the first phase of user testing, we sought out current participants (*n* = 15) in a tobacco cessation program, QuitCore, to help test the functionality and relevance of the tool (Phase 2). We engaged this particular group because they represented our target audience for the tool—namely tobacco users looking to quit. The participants were given a link to the Fact or Fiction tool prototype and were asked to complete the tool and take a short survey. The short 15–20 min open-ended survey focused on functionality, usability, and relevance (see Appendix B for the questionnaire). In collaboration with the coordinators of the QuitCore group, we conducted this second phase of user testing with 15 participants in November 2021. Results from the two phases of user testing were downloaded from RedCap and analyzed descriptively using SAS Studio version 9.4. Responses to long answers were thematically coded inductively using Excel software by one member of the research team (GJ-P).

### 2.3. Marketing

**Figure 2:**
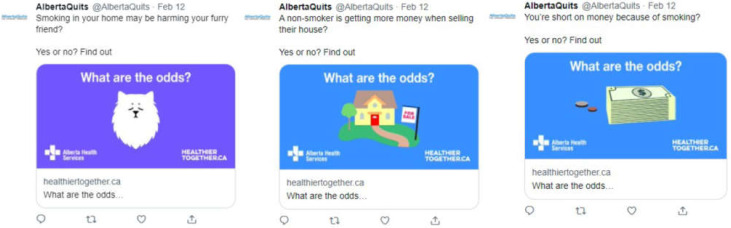
Examples of Single Images used in the Twitter Ad Campaign.

To promote this new tool, we placed an ad buy for a marketing campaign of the Fact or Fiction tool using the vendor, Kick Media. The marketing campaign lasted for 7 weeks (14 February 2022 to 1 April 2022) and used single images and animated graphics (see Figure 2) from the tool that were promoted across multiple social media channels (i.e., Facebook, Instagram, Snapchat, and Twitter). The target population for this marketing campaign was adults aged 18 or over who are currently using tobacco products, individuals looking to quit using tobacco products, and family members of people who use tobacco products across Alberta, Canada.

### 2.4. Evaluation: Reach

The Fact or Fiction Tobacco tool is housed on an AHS-dedicated self-hosted Canadian CACloud server. Non-identifiable data collected from users visiting the tool (e.g., tobacco use status, answers to Fact or Fiction questions, entry date, and postal code, if applicable) was stored on the CACloud server in a content management system (CMS). Data on whether individual users clicked on linked content in the tool (e.g., QuitCore or AlbertaQuits website links) are stored on Google Analytics. After 7 weeks (14 February–31 March 2022), data were extracted from these two sources and merged using a common ID number assigned to all non-bounced users of the Fact or Fiction tool. Data on individual users’ tobacco use status, postal code, and whether users clicked on suggested tobacco cessation resources were collected and used to analyze reach in this study.

#### 2.4.1. Inclusion/Exclusion Criteria

Users included in this sample were users who interacted with at least one question in the Fact or Fiction Tobacco tool. After interacting with a question, each user was assigned a user ID. No users with an ID were excluded from this analysis.

#### 2.4.2. Measures of Reach

The exposure variable of interest in this study was 3-level tobacco use status. This variable was generated based on responses to two questions at the beginning of the tool. The first question asks users “Do you currently use commercial tobacco or similar products (including e-cigarettes, vapes, dip, snus, cigarettes, cigars)?” If the user selected “no” to this question, they were classified as “non-tobacco users”, and if the user responded “yes” to this question, they were asked, “How do you feel about quitting?” There were three response options to this question: (1) I am not ready to quit, (2) I am thinking about quitting, and (3) I am making plans to quit. If the user selected “I am not ready to quit”, they were coded as “tobacco user not looking to quit”, and if the user selected response option 2 or 3, they were coded as “tobacco user looking to quit”.

The outcome variable of interest in this study was a binary variable (yes, no) of whether users clicked on any of the recommended supports and resources suggested to them based on their tobacco use status. Community or workplace tobacco cessation resources that encourage individuals to become smoke-free advocates in those settings were the resources suggested for non-tobacco users. Provincially funded tobacco cessation programs, including AlbertaQuits online and QuitCore, were the supports recommended for current tobacco users.

#### 2.4.3. Data Analysis

We analyzed the reach of the Fact or Fiction Tobacco tool using univariate and bivariate analyses after a period of 7 weeks. The period of data collection for this analysis spanned from the launch of the Fact of Fiction Tobacco tool on 14 February 2022 to 31 March 2022. To generate the reach indicators, we calculated the percent and frequency of users by tobacco use status. We conducted a bivariate analysis to determine the percent of clicks on resources by tobacco use status. In addition, we conducted a chi-square test to determine whether there were statistically significant differences in the percent of clicks on resources by tobacco use status using an alpha level of 0.05. Data were analyzed using SAS Studio version 9.4.

## 3. Results

### 3.1. User Testing: Informing Adaptation

Approximately 260 participants took our Phase 1 user testing survey and of these participants around 7% were current or occasional tobacco users and of that 7%, approximately 75% were looking to quit using tobacco. Based on the feedback from the first phase of user testing, several changes were made to the initial prototype, including muting the colour palette, incorporating animated visuals, alterations to text in the statements and inclusion of new suggested statements, and changes to reduce the amount of content and increase the readability of text. In addition, some notable changes included the inclusion of a widget at the end of the tool that allowed individuals to type in their postal code and receive recommendations for tobacco cessation resources in their area, including additional statements on the impact of quitting tobacco on family and finances, and allowing for use of the tool on phones and tablets.

A second phase of user testing was conducted with 15 current participants of QuitCore, a group tobacco cessation program offered in Alberta. Results from this second phase of user testing indicated that most of the users found the tool to be user-friendly and liked the overall presentation and layout of the tool. Approximately 50% of users agreed that they would recommend the tool to their friends and family who were currently using tobacco products. Participants of this second phase of user testing identified some areas for improvement such as loading speed, and some suggested statements for inclusion. These suggestions were incorporated into the final prototype ahead of the launch date in early 2022.

### 3.2. Marketing Campaign

The marketing campaign resulted in a click-through rate (i.e., the percent of users who click on an ad after seeing it) of 0.6% or almost 40,000 clicks across all platforms used (Facebook, Instagram, Twitter, Snapchat, Google). After receiving negative reactions to one of the images focused on the savings associated with quitting using tobacco, this image was removed from the marketing campaign on 17 March 2022. Most of the clicks on the marketing campaign on Facebook and Instagram (where age and gender data are available) came from those aged 65+ (23.4%) followed by those 35–44 years of age (21.1%) and those 45–54 years of age (20.7%). Women (52.5%) were slightly more likely to click than men (46%), and 1.5% of clicks came from those with unknown gender (see Table 1).

### 3.3. Evaluation: Reach

**Figure 3:**
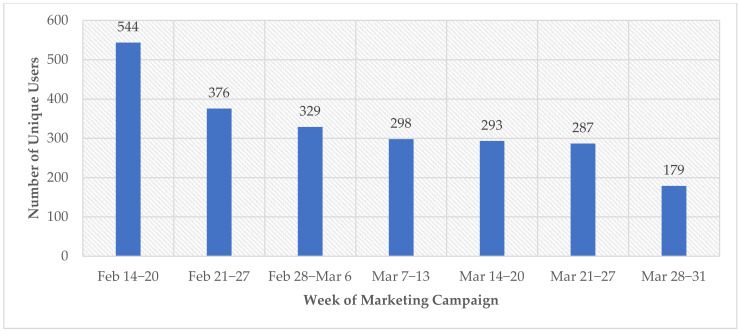
Number of users visiting the Tobacco Fact or Fiction tool per week for the 7-week marketing campaign (*n* = 2306).

After 7 weeks of data collection, 2306 users interacted with the Tobacco Fact or Fiction tool. Figure 3 below displays the number of users who interacted with the tool per week. The first week of the marketing campaign attracted the most users (*n* = 544) with a gradual decrease in interest over the duration of the campaign’s run.

Table 2 presents the percent and frequency of users of the tool by tobacco use status. Users were removed from this analysis if they had incomplete responses to the tobacco use questions (*n* = 62). Most of the users of the tool self-identified as non-tobacco users (61.2%), 29.7% of users were tobacco users looking to quit, and 9.1% of users were tobacco users who were not interested in quitting.

Table 3 presents the results from the bivariate analysis of clicks on suggested resources by tobacco use status. Overall, 9.8% of users, regardless of tobacco use status, clicked on suggested resources. We found statistically significant differences between the percent of users who clicked on suggested resources by tobacco use status. In total, 4.4% of non-tobacco users, 8.8% of tobacco users not looking to quit, and 21.2% of tobacco users looking to quit clicked on suggested resources.

## 4. Discussion

Through the development and implementation of the Fact or Fiction Tobacco virtual health tool, we aimed to inform, motivate, and support individuals in adopting positive health behaviour changes by accessing tobacco cessation resources. These resources may support users to quit using tobacco or support their community or workplace in becoming smoke-free. More specifically, the virtual health tool sought to correct prevailing myths about tobacco use and function as a mechanism for referral by assessing individuals’ readiness to quit and linking them with tailored content, resources, and interventions to aid them in their journey. Although simple in design, this tool has provided valuable insights into who is seeking tobacco cessation information, where they are at along the continuum of change, and whether they act by accessing the resources provided.

Insights from past research on virtual tool development emphasized the importance of conducting rigorous user testing to ensure ease of use for e-health tools [12]. The Fact or Fiction Tobacco tool underwent two phases of user testing with distinct populations to assess the content, look and feel, usability, and relevance of the tool. This two-stage user testing was followed by adaptations to improve the tool’s appearance and content relevance to the target population. The Fact or Fiction Tobacco tool reached its intended audience with almost 30% of users self-reporting current tobacco use; higher than the provincial prevalence of past 30-day use of tobacco products in 2019 of 13% [17]. This was likely aided by a marketing campaign that was targeted to reach current tobacco users across social media platforms. Therefore, to maintain engagement with the tool, a communication plan, including social media, radio, and print ads, will be developed in collaboration with our tobacco cessation partners.

Overall, the Fact or Fiction Tobacco tool has shown to be a successful way of reaching tobacco users looking to quit and connecting them to actionable resources that can help support them in making their goal a reality. These findings are supported by previously published articles that have shown that e-health tools can be effective in the identification of individuals in need of support, brief intervention or education, and referral [18,19]. Our virtual health tool accomplished this by identifying individuals looking to quit using tobacco in need of support, providing some brief education on myths around tobacco use and cessation tailored to their stage of change, and referring them to connect with resources available in their communities.

This study has several strengths. First, despite the growing number of virtual health tools being implemented globally, there are few published papers that describe the process of development and testing of the tools [13]. The Fact or Fiction Tobacco tool underwent two phases of user testing both at the population level and with the target population (i.e., tobacco users looking to quit). This allowed us to gain important insights into the useability of the tool across audiences. Second, the development of the tool was informed by health behaviour change theories that emphasized providing individuals with tailored information based on their intention to quit using tobacco products (i.e., stage of readiness for change). This theoretically informed approach is increasingly being recognized as an important step in creating effective virtual health tools [6,7], yet few studies published on the development of virtual health tools describe the evaluation of their outcomes in the context of their theory [20]. Our evaluation focused on examining outcome results based on these theory-informed stages of readiness for change. The RE-AIM framework for design and evaluation has been recognized as an effective way to evaluate e-health based on the review and synthesis of over 40 peer-reviewed and grey literature sources [21].

The present paper also has several limitations. First, the tool does not differentiate between repeat users. All users who interact with the screening questions are given a user ID. Yet, individuals looking to quit using tobacco often need multiple quit attempts to sustain long-term tobacco cessation [22]. Therefore, multiple visitors could be a result of multiple visits, but given the short time frame of this data collection, we do not think this to be the case or that this limitation may have inflated our reach. Second, the tool was not designed to track users’ interactions with the clicked-on resources beyond the initial click. Therefore, we do not have any information on what services or supports, if any, the individual viewed or signed up for, which limits our understanding of the nature of engagement that users will have with the actionable resources. However, this also presents an opportunity to collaborate in the future with local tobacco cessation programs to consider linking the data collected in the tool to the information collected in the tobacco cessation programs. Third, the information collected from this tool is self-reported and is therefore susceptible to bias due to a difference between the self-reported values and the true values.

## 5. Conclusions

In summary, the present paper described the theoretically informed and evidence-based development of a tobacco cessation tool that provided users with tailored content based on their readiness for change and linked individuals to actionable resources in their community. Two phases of user testing were conducted, and adaptations were made based on insights from both groups. Our tool was successful in reaching a larger than expected number of current tobacco users, and results from our analysis indicate that the tool was successful in getting individuals to access local resources to help them quit using tobacco products.

## Figures and Tables

**Table 1:** Percent Distribution of Facebook and Instagram Link Clicks by Gender and Age During the 7-week Marketing Campaign 14 February 2022–31 March 2022 (*n* = 8246 clicks).

Demographic Variable	Percent
65+ years	23.4%
55–64 years	16.8%
45–54 years	20.7%
35–44 years	21.1%
25–34 years	13.5%
18–24 years	4.5%
Women	52.5%
Men	46.0%
Unknown	1.5%

**Table 2:** Percent and Frequency Distribution of Users by Tobacco Use Status between 14 February 2022–31 March 2022 (*n* = 2244).

Tobacco Use Status	Percent	Frequency
Non-tobacco Users	61.2%	1375
Tobacco Users Not Looking to Quit	9.1%	204
Tobacco Users Looking to Quit	29.7%	666
Total	100%	2244

**Table 3:** Percent Chi-square Results of Users who Linked to Actionable Resources by Tobacco Use Status (*n* = 2244).

Tobacco Use Status	Percent	Frequency	*p*-Value
Non-tobacco Users	4.4%	61	<0.0001
Tobacco Users Not Looking to Quit	8.8%	18	
Tobacco Users Looking to Quit	21.2%	141	
All Users	9.8%	220	

## Data Availability

Data are not publicly available and not available by request.

## Appendix A. Questionnaire for Phase 1 User Testing

**Table:**

Question #	Fact or Fiction User Testing: Phase One
Intro	Please review the Fact or Fiction Tool at the following link and then return to this survey to answer the questions that focus on the content and “look and feel” of the Tool. Please note that the design of the tool is still underway and several features (e.g., sliders and animation) are not active. There will be notes throughout the tool indicating how the final design will look. It should take you approximately 15–20 min to review the tool and complete this survey. This project has ethics approval through A Project Ethics Community Consensus Initiative (ARECCI). All your answers will be kept private and confidential and will not be linked to your name or contact information. If you have any questions or concerns, please contact Dr. Lisa Allen Scott (lisa.allenscott@ahs.ca). Please review the Fact or Fiction tool by visiting the following link and then return to this survey to answer the questions that focus on the content and “look and feel” of the tool.
1	Have you reviewed the Fact or Fiction tool? Yes No
2	Do you currently use commercial tobacco or similar products (including e-cigarettes, vapes, dip, snus, cigarettes, cigars)? Yes (if selected, proceed to Q3) No (if selected, skip to Q4)
3	How do you feel about quitting? (any selection skip to Q6) I’m not ready to quit I’m thinking about quitting sometime I’m making plans to quit soon
4	Are your friends and/or family looking to quit using tobacco? Yes No
5	How likely are you to recommend the Fact or Fiction tool to family or friends who are using tobacco products? (any selection skip to Q10) Very likely Likely Unlikely Very unlikely Not sure
6	How likely are you to connect with the supports and programs presented in the Fact or Fiction tool? Very likely Likely Unlikely Very unlikely Not sure
7	Would you rather see AlbertaQuits resources and links on every answer page or only at the end of the tool? Every answer page Only at the end of the tool Not sure Other, please explain
8	How likely are you to include your postal code to be connected with local resources to help you quit using tobacco products? Very likely Likely Unlikely Very unlikely Not sure
9	How likely are you to include your email address to be contacted by a certified tobacco cessation coach to help you quit using tobacco products? Very likely Likely Unlikely Very unlikely Not sure
10	What did you like most about the Tobacco Fact or Fiction tool? [open text response]
11	What did you like least about the Tobacco Fact or Fiction tool? [open text response]
12	Are there any statements/content in the Tobacco Fact or Fiction tool that you found confusing or offensive? [open text response]
13	What suggestions do you have for improving the Tobacco Fact or Fiction tool? [open text response]
14	What parts of the Tobacco Fact or Fiction tool should stay the same? [open text response]

## Appendix B. Questionnaire for Phase 2 User Testing

**Table:**

Question #	Fact or Fiction User Testing: Phase Two
Intro	Please review the Tobacco Fact or Fiction Tool at the link provided to you by email from your QuitCore facilitator and then return to this survey to answer the questions that focus on the functionality of the Tool. It should take you approximately 15–20 min to review the Tobacco Fact or Fiction tool and complete this survey. This project has ethics approval through A Project Ethics Community Consensus Initiative (ARECCI). All your answers will be kept private and confidential and will not be linked to your name or contact information. If you have any questions or concerns, please contact Dr. Lisa Allen Scott (lisa.allenscott@ahs.ca). Please review the Fact or Fiction Tool by visiting the following link and then return to this survey to answer the questions that focus on the functionality of the Tool.
1	Have you reviewed the Fact or Fiction tool? Yes No
DEVICE FUNCTIONALITY	
2	What type of device did you use to review the Fact or Fiction tool (select all that apply)? Laptop Desktop Mobile phone Tablet Other, please specify
3	Please enter the Internet Browser (e.g., chrome, explore, edge, safari) that you used to review the Fact or Fiction tool. [open text response]
4	Did you have any issues using the Fact or Fiction tool on any of the devices or browsers indicated above? If yes, please describe. [open text response]
DEVICE USABILITY	
5	I thought the Tobacco Fact or Fiction tool was user-friendly. Strongly Disagree Disagree Neutral Agree Strongly Agree
6	I like the overall presentation of the Tobacco Fact or Fiction tool. Strongly Disagree Disagree Neutral Agree Strongly Agree
7	I like the overall layout of the Tobacco Fact or Fiction tool. Strongly Disagree Disagree Neutral Agree Strongly Agree
8	I was easily able to complete the Tobacco Fact or Fiction tool. Strongly Disagree Disagree Neutral Agree Strongly Agree
9	I am likely to recommend the Tobacco Fact or Fiction tool to family and/or friends who are using tobacco products. Strongly Disagree Disagree Neutral Agree Strongly Agree
10	It was clear where to click to connect to local resources in the Tobacco Fact or Fiction tool. Strongly Disagree Disagree Neutral Agree Strongly Agree
11	Please provide any additional feedback on your experience using the Tobacco Fact or Fiction tool (i.e., design, navigation, likes and dislikes) and elaborate on problems and provide suggestions for improvement. [open text response]
RELEVANCE	
12	What did you like most about the Tobacco Fact or Fiction tool? [open text response]
13	What did you like least about the Tobacco Fact or Fiction tool? [open text response]
14	Was your motivation to quit using commercial tobacco captured in any of the statements in the Fact or Fiction tool? If yes, please describe.If no, what was your motivation to quit? [open text response]
15	Were the online resources that the Tobacco Fact or Fiction tool connected you to after entering your postal code useful? Please describe what services or resources were presented to you and which ones would be valuable/not valuable. [open text response]
16	What else would you like to tell us about the Tobacco Fact or Fiction tool that could help us improve the user experience? [open text response]
